# Does the Uterine Injection Site Matter for the Pelvic Sentinel Lymph Node Mapping? A Systematic Review and Meta-Analysis

**DOI:** 10.3390/medicina61040699

**Published:** 2025-04-10

**Authors:** Pier Carlo Zorzato, Simone Garzon, Mariachiara Bosco, Filippo Ferrari, Francesca Magni, Rosa Maria Laterza, Antonio Simone Laganà, Francesco Fanfani, Stefano Uccella

**Affiliations:** 1 Unit of Obstetrics and Gynecology, Department of Surgery, Dentistry, Pediatrics, and Gynecology, AOUI Verona, University of Verona, 37125 Verona, Italy; piercarlo86.pcz@gmail.com (P.C.Z.); simone.garzon@univr.it (S.G.); mariachiara.bosco@univr.it (M.B.); ferrarifilippoalberto@gmail.com (F.F.); magnifrancesca@outlook.it (F.M.); 2 Division of General Gynecology and Gynecologic Oncology, Department of Obstetrics and Gynecology, Medical University of Vienna, 1090 Wien, Austria; Karl Landsteiner Society for Special Gynecology and Obstetrics, 3100 St. Pölten, Austria; rosa.laterza@meduniwien.ac.at; 3 Unit of Obstetrics and Gynecology, Department of Health Promotion, Mother and Child Care, Internal Medicine and Medical Specialties (PROMISE), “Paolo Giaccone” Hospital, University of Palermo, 90127 Palermo, Italy; antoniosimone.lagana@unipa.it; 4 Gynecologic Oncology Unit, Department of Women, Children and Public Health Sciences, Fondazione Policlinico Universitario Agostino Gemelli IRCCS, 00168 Rome, Italy; francesco.fanfani@policlinicogemelli.it; 5 Catholic University of the Sacred Heart, 00168 Rome, Italy

**Keywords:** sentinel lymph node, pelvis, uterus, lymphatic pathway, gynecological cancers

## Abstract

*Background and Objectives*: To summarize the evidence on in vivo uterine pelvic lymphatic drainage. *Materials and Methods*: A literature search was performed in multiple electronic databases from inception to December 2024. We included all the studies that compared two different uterine injection sites in the mapping of pelvic sentinel lymph nodes by injecting two different tracers into two distinct injection sites. The primary outcomes included the concordance and discordance rates in the mapped pelvic sentinel lymph nodes between the pairs of injection sites. The secondary outcomes were the detection rates per injection site and tracer. Four reviewers independently reviewed the records for inclusion, assessed the risk of bias, and extracted the data. Pooled concordance, discordance, and detection rates with 95% confidence intervals (CIs) were estimated using the random effects model. Heterogeneity was quantified using the I^2^ tests. *Results*: Out of 2512 records, we included 4 studies (172 patients and 344 hemipelves). Three studies injected the cervix with the technetium-99m and the uterine corpus with methylene blue; one study injected the cervix with indocyanine green and the utero-ovarian ligament with methylene blue. Both tracers/injection sites successfully identified a sentinel lymph node in 132 hemipelves (132/344; 38.4%), identifying the same sentinel lymph node in 116 cases (116/132; 87.9%). The pooled concordance rate per hemipelvis was 91.8% (95% CI 0.665–1.000; I^2^ = 92%; chi^2^ *p*-value < 0.01). Two different sentinel lymph nodes were identified in the remaining 16 hemipelves, with a pooled hemipelvis discordance rate of 8.2% (95% CI 0.000–0.335; I^2^ = 92%; chi^2^ *p*-value < 0.01). The cervix and technetium-99m were the injection site and tracer with the highest pooled detection rate. *Conclusions*: Different uterine injection sites appear to share a common pelvic lymphatic pathway and sentinel lymph node in most cases, consistent with the current practice in endometrial cancer. Future research will confirm whether cervical injections might be proposed for pelvic sentinel lymph node mapping in all gynecological cancers.

## 1. Introduction

Sentinel lymph node mapping algorithms have been integrated into the staging of most gynecological cancers [1,2]. Only in apparent early-stage ovarian neoplasm is this technique still debated [3,4,5].

Sentinel lymph node mapping algorithms are based on the anatomy of the lymphatic system and the expected drainage pathways from the genital organs [6,7], directly affecting the choice of anatomical sites for tracer injection. However, evidence regarding the actual in vivo lymphatic drainage is limited [8,9]. Specifically, whether different anatomic sites of tracer injection drain differently or equally in vivo, identifying a different versus the same sentinel lymph node is not clear for all possible alternative injection sites.

Testing whether two different tracer injection sites map to the same or a different sentinel lymph node is relevant when the most intuitive method employs a more complex and less reliable technique. In endometrial cancer, the cervical injection has been compared with and favored over hysteroscopic peritumoral or uterine corpus tracer injections because they were equally effective yet easier [10]. A similar issue has been raised for sentinel lymph node mapping in apparent early-stage ovarian cancer. Observed poor pelvic detection rate raises concerns about the suitability of the utero-ovarian ligament as an appropriate uterine injection site for pelvic sentinel lymph node mapping [4,11]. Technical issues and possible inefficient pelvic lymphatic drainage, particularly in delayed staging with previous adnexectomy [5], highlights the need to use other injection sites [4].

Based on this, an in-depth understanding of the uterine pelvic lymphatic drainage system in vivo is paramount for pelvic sentinel lymph node mapping in apparent early-stage ovarian cancer [4]. Therefore, we conducted a systematic review and meta-analysis of the studies that have investigated uterine pelvic lymphatic drainage by injecting two different tracers into two distinct uterine injection sites. The use of two tracers in two distinct uterine injection sites enables a direct comparison in the same patient and clarifies whether the identified pelvic SLN is the same or different. This evidence may be crucial in supporting or disputing the use of different injection sites than the specific tumor location for pelvic SLN mapping in gynecological cancers (i.e., the cervix in endometrial cancer and potentially in ovarian cancer).

## 2. Materials and Methods

The systematic review and meta-analysis was planned before beginning the online search and conducted in accordance with the Cochrane Handbook for Systematic Reviews of Diagnostic Test Accuracy [12]. The findings were reported following the Preferred Reporting Items for Systematic Reviews and Meta-Analyses of Diagnostic Test Accuracy (PRISMA-DTA) [13]. The protocol has been registered on PROSPERO (CRD 42024612435).

### 2.1. Search Strategy and Eligibility Criteria

A certified professional librarian (Biblioteca Meneghetti—Verona University) performed a literature search, from database inception to December 2024, in the following electronic databases: EMBASE, Scopus, PubMed, Web of Science, and the Cochrane Library. The search strategy included combinations of the following keywords: “ovarian cancer”, “endometrial cancer”, “cervical cancer”, “Indocyanine green”, “Methylene blue”, “Technetium 99”, “Lymph Node Metastasis”, “sentinel lymph node”, and “injection”. The detailed search strategy is available in the Supplementary Materials. The references of all included records were systematically revised.

We included all the studies meeting the predetermined inclusion criteria. The population comprised patients with gynecological cancer undergoing pelvic sentinel lymph node mapping. For the index test, pelvic sentinel lymph node mapping was performed by injecting a tracer into a specific site along the female genital tract; for the comparator test, a different tracer was injected into a second different site along the female genital tract. The primary outcomes were the concordance and discordance rates in the pelvic sentinel lymph nodes mapped per hemipelvis between the pairs of injection sites; the secondary outcomes were injection-site-specific and tracer-specific detection rates. We excluded any records that did not present original study data. No language exclusion criterion was applied; non-English records were translated with Google Translator [14].

### 2.2. Study Selection and Data Extraction

After duplicate removal, two reviewers independently screened the titles and abstracts of the identified records (PCZ, FFA). Two other reviewers (SG, FM) retrieved and independently assessed the full text of potentially eligible publications. Any disagreement was resolved with a further reviewer (SU). Two reviewers (PCZ, SG) developed and used a standardized form to extract the following data from the selected studies: authors, publication year, research setting, study design, patient population (i.e., age, number of patients, and types, histotype, and stages of cancer), sites of injection, injection techniques, types of tracers, adverse events, outcome measures (hemipelvis level data), and items for quality evaluation.

### 2.3. Assessment of Risk of Bias

Two reviewers (FFA and PCZ) independently assessed the risk of bias in the selected studies following a modified version of the Quality Assessment of Diagnostic Accuracy Studies tool 2 (QUADAS-2) (Supplementary Materials). Any disagreement was resolved with a further reviewer (SG) [15].

### 2.4. Data Synthesis

The concordance rate was defined as the number of hemipelves with the same sentinel lymph node mapped by both tracers out of the total number of hemipelves with double successful mapping. The discordance rate was defined as the number of hemipelves with two different sentinel lymph nodes mapped by the two tracers out of the total number of hemipelves with double successful mapping. The concordance and discordance rates were calculated per pair of injection sites. The detection rate was defined as the number of hemipelves in which at least one sentinel lymph node was successfully identified out of the total number of hemipelves, according to the injection site and tracer.

Proportion meta-analyses were conducted using the random effects model to estimate the pooled proportions with 95% confidence intervals (CIs) for the concordance and discordance rates per pair of injection sites and detection rates per injection site and tracer. Heterogeneity was quantified using the I² tests; an I² less than 25% was considered low, and an I² more than 75% was considered high. When ten or more studies were identified, publication bias was assessed by funnel plot and Egger test. All analyses were two-tailed with a statistical significance threshold of *p* < 0.05. R Statistical Software (v4.1.2; R Core Team 2024, Vienna, Austria) was used.

## 3. Results

### 3.1. Study Selection

The initial literature search retrieved 2512 records, including a cross-reference review. After duplicate removal, 1491 records underwent title and abstract screening, with 34 articles that underwent a full-text assessment. Thirty studies were excluded for two reasons. Most studies were excluded because they investigated a single injection site or tracer. When two uterine injection sites were analyzed, the same tracer was used, or two different tracers were injected into the same uterine site. A minority did not provide the outcome of interest; although two different tracers were injected into two different uterine sites, the authors did not provide the concordance or discordance rate. Therefore, four records regarding four different studies were finally included (Table 1) [16,17,18,19]. The PRISMA Flowchart illustrates the study selection process (Figure S1).

### 3.2. Study Characteristics

The four studies encompassed 172 patients who underwent pelvic sentinel lymph node mapping for endometrial cancer (Table 1). Three studies injected the cervix with technetium-99m and the uterine corpus with methylene blue [17,18,19]; one study injected the cervix with indocyanine green and the utero-ovarian ligament with methylene blue [16]. In all four studies, the cervical injection was compared with an alternative injection site. The data on sentinel lymph node mapping are reported in Table 2.

### 3.3. Risk of Bias Assessment

Two studies were considered at high risk of bias in the patient selection domain because of the exclusion of cases with suspicious lymph nodes on preoperative imaging [18,19]. One study failed to provide details regarding the patient recruitment method, so the selection domain was considered unclear [17]. One study was deemed unclear in the reference standard domain because of missing detailed clinical data [18] (Figure S2, Table S1).

### 3.4. Concordance and Discordance Rates

**Figure 1:**
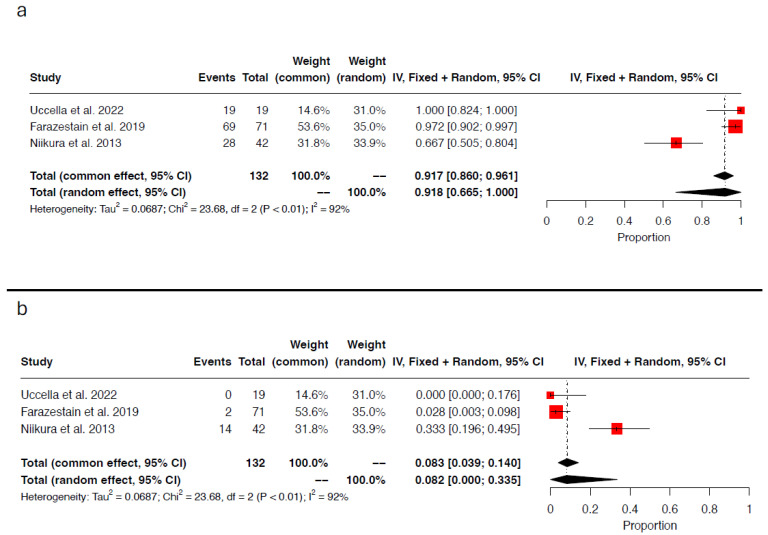
Forest plot of proportional meta-analysis for (**a**) concordance rate per hemipelvis and (**b**) discordance rate per hemipelvis [16,17,18].

Both tracers/injection sites successfully identified of at least one sentinel lymph node in 132 hemipelves (132/344; 38.4%) and the same sentinel lymph node in 116 out of 132 hemipelvis (116/132; 87.9%). The pooled concordance rate per hemipelvis was 91.8% (95% CI 0.665–1.000; I^2^ = 92%; chi^2^ *p*-value < 0.01) (Figure 1a). Two different sentinel lymph nodes were detected in the remaining 16 hemipelves, leading to a pooled hemipelvis discordance rate of 8.2% (95% CI 0.000–0.335; I^2^ = 92%; chi^2^ *p*-value < 0.01) (Figure 1b).

### 3.5. Detection Rate per Injection Site

**Figure 2:**
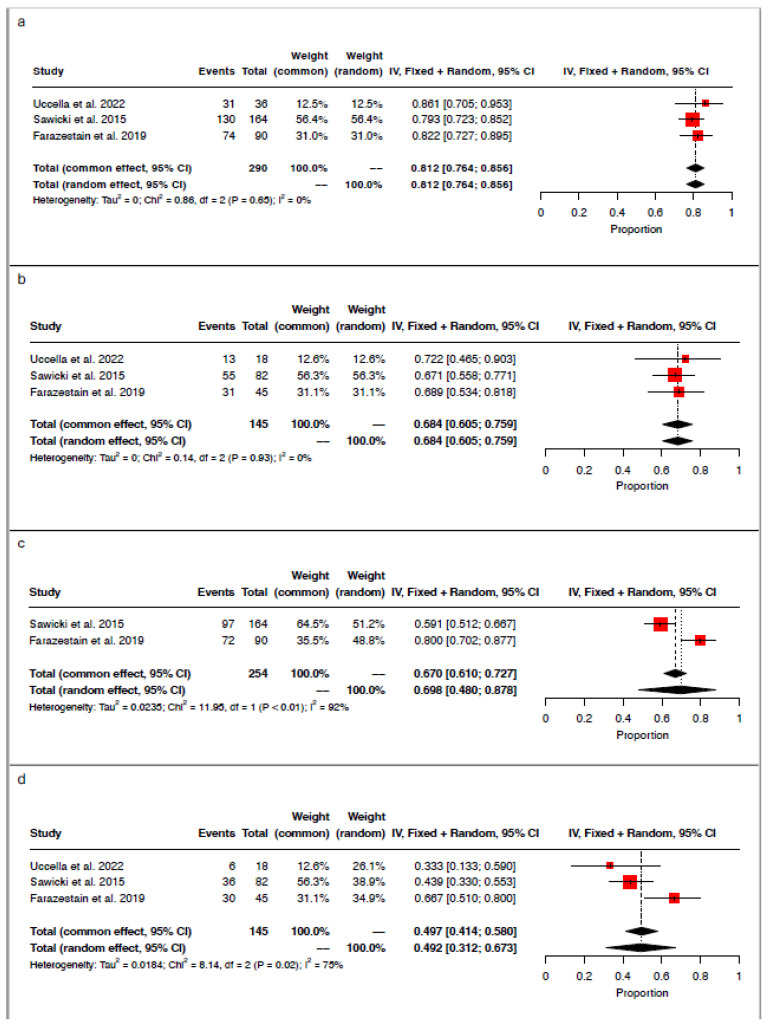
Forest plot of proportional meta-analysis for (**a**) hemipelvis detection rate for cervical injection, (**b**) bilateral detection rate for cervical injection, (**c**) hemipelvis detection rate for uterine corpus injection, and (**d**) bilateral detection rate for uterine corpus injection [16,17,19].

The pooled detection rate for the cervical injection (technetium-99m or indocyanine green) was 81.2% (95% CI 0.764–0.856; I^2^ = 0%; chi^2^ *p*-value = 0.65), with 235/290 hemipelves with one identified sentinel lymph node (Figure 2a). Bilateral mapping was achieved in 99/145 patients, leading to a pooled bilateral detection rate for cervical injection of 68.4% (95% CI 0.605–0.759; I^2^ = 0%; chi^2^ *p*-value = 0.93; Figure 2b).

Regarding the uterine corpus injection (methylene blue), the pooled hemipelvis detection rate was 69.8% (95% CI 0.480–0.878; I^2^ = 92%; chi^2^ *p*-value < 0.01; Figure 2c), with 169/254 hemipelves having an identified sentinel lymph node. Bilateral mapping was achieved in 72/145 cases, with a pooled bilateral detection rate of 49.2% (95% CI 0.312–0.673-; I^2^ = 75%; chi^2^ *p*-value = 0.02; Figure 2d).

Only 18 patients from the study by Uccella et al. [16] underwent a tracer injection into the utero-ovarian ligament (methylene blue). At least one sentinel lymph node was identified in 19 out of 36 hemipelves, with a detection rate of 52.8% and a bilateral detection rate of 33.3% [16].

### 3.6. Detection Rate per Tracer

**Figure 3:**
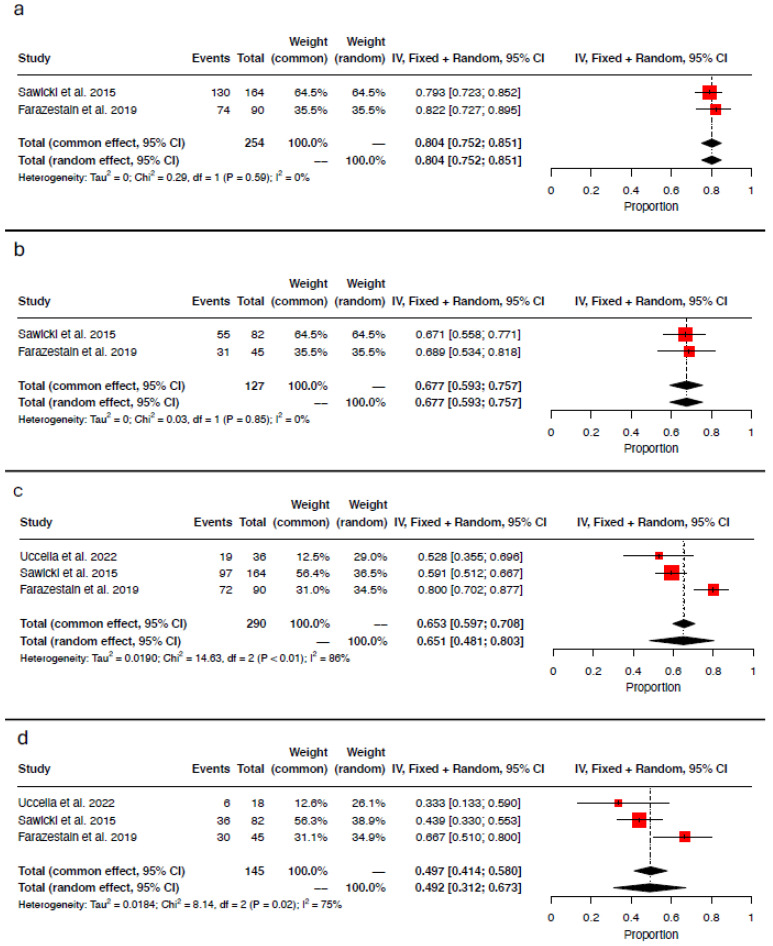
Forest plot of proportional meta-analysis for (**a**) hemipelvis detection rate for technetium-99m, (**b**) bilateral detection rate for technetium-99m, (**c**) hemipelvis detection rate for methylene blue, and (**d**) bilateral detection rate for methylene blue [16,17,19].

Only two authors reported results on technetium-99m alone (cervix) [17,19]. According to these findings, 204/254 hemipelves had at least one detected sentinel lymph node (pooled detection rate 80.4%; 95% CI 0.752–0.851; I^2^ = 0%; chi^2^ *p*-value = 0.59; Figure 3a). Bilateral mapping was achieved in 86/127 patients, with a pooled Technetium-99m bilateral detection rate of 67.7% (95% CI 0.593–0.757; I^2^ = 0%; chi^2^ *p*-value = 0.85; Figure 3b). Data regarding the injection of methylene blue alone (uterine corpus or utero-ovarian ligament) were available from three authors [16,17,19]. At least one sentinel lymph node was detected in 188/290 hemipelves (pooled methylene blue detection rate 65.1%; 95% CI 0.481–0.803; I^2^ = 86%; chi^2^ *p*-value <0.01; Figure 3c). Bilateral mapping was accomplished in 72/145 individuals, resulting in a pooled methylene blue bilateral detection rate of 49.2% (95% CI 0.312–0.673; I^2^ = 75%; chi^2^ *p* value = 0.02; Figure 3d). Indocyanine green (cervix) was employed exclusively in the study by Uccella et al. [16], with a detection rate of 86.1% per hemipelvis and a bilateral detection rate of 72.2%.

## 4. Discussion

All studies that investigated pelvic sentinel lymph node mapping by injecting two different tracers into two distinct injection sites compared the cervical injection with the uterine corpus or the utero-ovarian ligament, and the pooled concordance rate in the identified pelvic sentinel lymph node was high. The cervix and technetium-99m reported the highest detection rates.

The studies investigating uterine corpus/perilesional versus cervical injections for pelvic sentinel lymph node mapping in endometrial cancer were based on the assumption that different injection sites would determine different pelvic sentinel lymph nodes. An injection site distant from the tumor may be less valid, not reflecting the tumoral lymphatic drainage [20,21,22,23]. In opposition to this assumption, the pooled high concordance rate suggests that a dominant uterine lymphatic drainage pathway and associated sentinel lymph node may be present in most female pelves. In most cases, the two tracers identified the same pelvic sentinel lymph node regardless of the injection site. This is consistent with the current practice and available evidence in endometrial cancer [24]. The studies that compared the cervical versus hysteroscopic peritumoral or uterine corpus injections in endometrial cancer did not observe differences in the anatomical distribution of the pelvic sentinel lymph nodes [10,20,21]. Furthermore, the pelvic sentinel lymph node location was not observed to be associated with the endometrial cancer location in the uterine cavity [25].

Nevertheless, in a minority of cases, the pelvic sentinel lymph node identified differed between the two injection sites, and only one of the two would have been identified with the standard practice, resulting in possible suboptimal staging. This observation potentially questions the use of an injection site different from the tumor site for all gynecological cancers. However, caution is needed when interpreting the pooled discordance rate. Two different homolateral pelvic sentinel lymph nodes for the uterus do not imply suboptimal staging. Identifying two pelvic sentinel lymph nodes on the same side is common; more efficient tracers with adequate waiting time may identify both sentinel lymph nodes. In the FILM trial, a mean number of 3.2 (1.6) pelvic sentinel lymph nodes per patient was obtained [26]. Additionally, the pooled discordance rate was characterized by high heterogeneity. The study by Niikura et al. provided a discordance rate higher than the other studies, raising concerns on whether study-specific factors may have increased the discordance rate. A sensitivity analysis excluding Niikura et al. showed higher concordance (98.5%; 95% CI 0.94–1.000; I^2^ = 0%) and lower discordance (2.8%; 95% CI 0.001–0.079; I^2^ = 0%) rates with low heterogeneity. The high concordance and low discordance rates support the hypothesis that two different uterine injection sites may detect the same pelvic SLN, allowing the choice of the injection site to be based on other factors. These observations are consistent with the current use of the cervical injection as a substitute for the more precise injection at the tumor level in endometrial cancer [20,21,24].

The pooled detection rate was higher for technetium-99m than methylene blue. Indocyanine green was only used in one study, and meta-analysis was not feasible. These results are consistent with the current practice and available evidence showing methylene blue as the tracer with the lowest performance [26,27,28,29]. In this regard, differences between the tracers may explain the discrepancies between injection sites, as methylene blue was constantly used for the injection site alternative to the cervix.

However, the advantage of using a cervical injection in mapping the pelvis over other uterine injection sites has already been observed. In most investigations, the pelvic sentinel lymph node detection rate was higher with the cervical injection of indocyanine green than with a perilesional hysteroscopic/uterine corpus injection [10,20]. The risk of tracer spillage and the lower injected volume associated with a hysteroscopic injection are technical issues that may increase the risk of failure and the need for experienced surgeons [10,20]. Technical issues and a lower pelvic lymphatic drainage efficiency may explain the similarly low pelvic sentinel lymph node detection rate observed for the utero-ovarian ligament. The most comprehensive meta-analysis on sentinel lymph node mapping in apparent early-stage ovarian cancer reported a pelvic detection rate of 59.4% (66/111) [4]. Notably, Rey et al. estimated the pelvic detection rate, by utero-ovarian ligament injection, only among 111 out of 239 total cases (46.4%). Most cases were excluded due to protocol violations in the utero-ovarian ligament injection, which were reported in up to 69.8% of cases in the SELLY trial [4,11,30]. Laven et al., who focused on patients who underwent restaging after adnexectomy, provided a pelvic detection rate of 27% [31]. On these bases, the question of whether cervical injections may be equal to but more efficient and straightforward than utero-ovarian ligament injections for sentinel lymph node mapping in apparent early-stage ovarian cancer merits further investigation [4]. Unlike other studies [11,31], Lago et al. reported a higher detection rate of 93% [32]. This difference may be attributed to the deeper injection of the tracer near the dorsal/lateral parametrium rather than in the utero-ovarian stump [11].

This is the first systematic review and meta-analysis that specifically investigated whether using two different injection sites of the uterus would map the same or a different pelvic SLN. The rigorous and systematic approach with strict adherence to the predetermined criteria strengthens the study results. The selective inclusion of studies in which two different uterine injection sites were investigated using two different tracers in the same patient allowed for an in vivo anatomical investigation of the uterine lymphatic drainage system with a direct comparison between the injection site pairs. However, identifying only four studies involving 172 patients limits the study results. Moreover, only studies enrolling patients with endometrial cancer were included; therefore, caution is needed when inferring hypotheses on other gynecological malignancies. Additionally, indocyanine green was underrepresented. Considering indocyanine green as the standard, the results provided can be regarded as outdated. However, the tracer and underlying cancer are unlikely to represent confounders for the concordance rate. Furthermore, none of the trials randomized the tracer to the two injection sites, and only one study was designed for the anatomical investigation of uterine lymphatic drainage [16]. These characteristics determine that the results are often secondary outcomes, and that each injection site was linked to a specific tracer, thereby connecting the two performances. Moreover, the inclusion of two studies with a high risk of bias in the patient selection domain, due to the exclusion of cases with suspicious lymph nodes on preoperative imaging, raises concerns about generalizability. Finally, the identification of only four studies also impeded investigations into the presence of publication bias.

Understanding the pelvic lymphatic drainage of the uterus impacts the choice of the tracer injection site for pelvic sentinel lymph node mapping [6,7]. Clarifying whether two different uterine sites for tracer injection drain differently or equally in the pelvis becomes essential, especially given that the more intuitive peritumoral injection is a more complex technique with a high failure rate and low detection rate. Our results suggest that, in most patients, a main pelvic lymphatic drainage pathway and a common sentinel lymph node are present regardless of the injection site. These observations are consistent with the available evidence and support the current practice in endometrial cancer [24]. Moreover, our results may have implications for pelvic sentinel lymph node mapping in apparent early-stage ovarian cancer, supporting further investigations into whether the cervical injection may be equal but more efficient than the utero-ovarian ligament injection.

## 5. Conclusions

Our findings support the hypothesis that different uterine pelvic lymphatic pathways share a common pelvic sentinel lymph node in most cases. These observations support the current clinical practice in endometrial cancer and have significant implications on sentinel lymph node mapping in apparent early-stage ovarian cancer. The cervical injection of indocyanine green, with advantages in terms of accessibility and reliability, might be proposed in future investigations into pelvic sentinel lymph node mapping in apparent early-stage ovarian cancer.

## Figures and Tables

**Table 1:** Characteristics of studies included.

Author, Year (Country)	Design	N of Patients	Histotype	Stage (FIGO)	Lymphovascular Invasion	Surgical Approach	Site of Injection	Tracer	Technique	Time Tracer Injection—SLN Evaluation
Niikura et al., 2013 [18] (Japan)	Prospective	27	NA	NA	NA	Open Surgery	Cervix	99mTc	Pre-operative lymphoscintigraphy by injecting Tc99m-labeled phytate in the cervix at 3, 6, 9, and 12 o’clock positions one day before surgery. At the time of surgery, a gamma-detecting probe was used to locate radioactive SLN.	NA
							Corpus	MB	Injection into five different sites of the subserosal endometrium.	
Sawicki et al., 2015 [19] (Poland)	Retrospective	82	96.4% endometrioid 1.2% clear cell 2.4% serous	IA: 47.6% IB: 23.2% II: 17.1% IIIA: 2.4% IIIB: 2.4% IIIC1: 7.3%	Yes: 12.2% No: 87.8%	Open Surgery	Cervix	99mTc	Injection of the cervix with Tc99m-labeled nanocolloid and the SLN was subsequently located using a handheld gamma probe.	NA
							Corpus	MB	4 mL in the uterine fundus.	
Farazestanian et al., 2019 [17] (Iran)	Prospective	45	86.7% endometrioid 8.9% clear cell 4.4% serous	NA	Yes: 15.6% No: 84.4%	Open Surgery	Cervix	99mTc	Two injections of Tc99m-phytate into the cervix at the 6- and 12-o’clock positions the day before surgery. Planar lymphoscintigraphy was conducted for each patient 8 to 18 h after injections, and surgery was scheduled 18 to 24 h after the radiotracer injection. Hot SLN were identified using a handheld gamma probe.	15 min
							Corpus	MB	2 mL at subserosal fundal midline locations	
Uccella et al., 2022 [16] (Italy)	Prospective	18	94.5% endometrioid 5.5% non endometrioid	IA: 38.8% IB: 44.4% II: 11.1% IIIC: 5.6%	Yes: 55.5% No: 44.5%	Conventional Laparoscopy	Cervix	ICG	2 mL ICG at 3 and 9 o’clock positions, 1 mL deeply at 1.5–2.5 cm into the stroma, and 1 mL superficially into the submucosal tissue.	15 min
							UOL	MB	2 mL bilateral injections into the utero-ovarian ligaments trans-abdominally under laparoscopic vision. Nodes were detected using a near-infrared high-intensity light source.	

SLN: Sentinel lymph node; N: Number; UOL: Utero-ovarian ligament; 99mTc: Technetium-99m; MB: Methylene blue; ICG: Indocianine green; NA: Not available; FIGO: International Federation of Gynecology and Obstetrics.

**Table 2:** Concordance and detection rates in included studies.

Author, Year	N of Patients/Hemipelvis	Site of Injection	Tracer	Detection Rate			Hemipelvis with Double Mapping	Concordance Rate	Discordance Rate
				Mapped Hemipelvis	Patients with at Least One Mapped Hemipelvis	Patients with Bilateral Pelvic Mapping			
Niikura, 2013 [18]	27/54	Cervix	99mTc	NA	NA	NA	42	28/42 (66.7%)	14/42 (33.3%)
		Corpus	MB	NA	NA	NA			
		Cervix + Corpus		42/54 (77.8%)	27/27 (100%)	26/27 (96.3%)			
Sawicki, 2015 [19]	82/164	Cervix	99mTc	130/164 (79.3%)	75/82 (91.5%)	55/82 (67.1%)	NA	NA	NA
		Corpus	MB	97/164 (59.1%)	61/82 (74.4%)	36/82 (43.9%)			
		Cervix + Corpus		140/164 (85.4%)	78/82 (95.1%)	62/82 (75.6%)			
Farazestanian, 2019 [17]	45/90	Cervix	99mTc	74/90 (82.2%)	42/45 (93.3%)	31/45 (68.8%)	71	69/71 (97.2%)	2/71 (2.8%)
		Corpus	MB	72/90 (80.0%)	42/45 (93.3%)	30/45 (66.6%)			
		Cervix + Corpus		76/90 (84.4%)	42/45 (93.3%)	43/45 (95.5%)			
Uccella, 2022 [16]	18/36	Cervix	ICG	31/36 (86.1%)	18/18 (100%)	13/18 (72.2%)	19	19/19 (100%)	0/19 (0%)
		UOL	MB	19/36 (52.8%)	13/18 (72.2%)	6/18 (33.3%)			
		Cervix + UOL		31/36 (86.1%)	18/10 (100%)	13/18 (72.2%)			

N: number; DR: detection rate; UOL: utero-ovaria ligament; 99mTc: Technetium-99m; MB: methylene blue; NA: not available.

## Supplementary Materials

The following supporting information can be downloaded at: https://www.mdpi.com/article/10.3390/medicina61040699/s1, Figure S1: PRISMA flowchart of study selection; Figure S2: Risk of Bias assessment with the Modified Quality Assessment of Diagnostic Accuracy Studies tool 2 (QUADAS-2); Table S1: Risk of bias assessment for each included study based on the Modified Quality Assessment of Diagnostic Accuracy Studies tool 2 (QUADAS-2).
