# Supplementary material for: Does the Uterine Injection Site Matter for the Pelvic Sentinel Lymph Node Mapping? A Systematic Review and Meta-Analysis
Source: Medicina (Kaunas). 2025 Apr 10;61(4):699. doi: 10.3390/medicina61040699 (PMC12028796; doi:10.3390/medicina61040699)
Supplement: Supplementary file 1 [file medicina-61-00699-s001.zip › Figure_S1.pdf]

Figure S1. PRISMA flowchart of study selection.

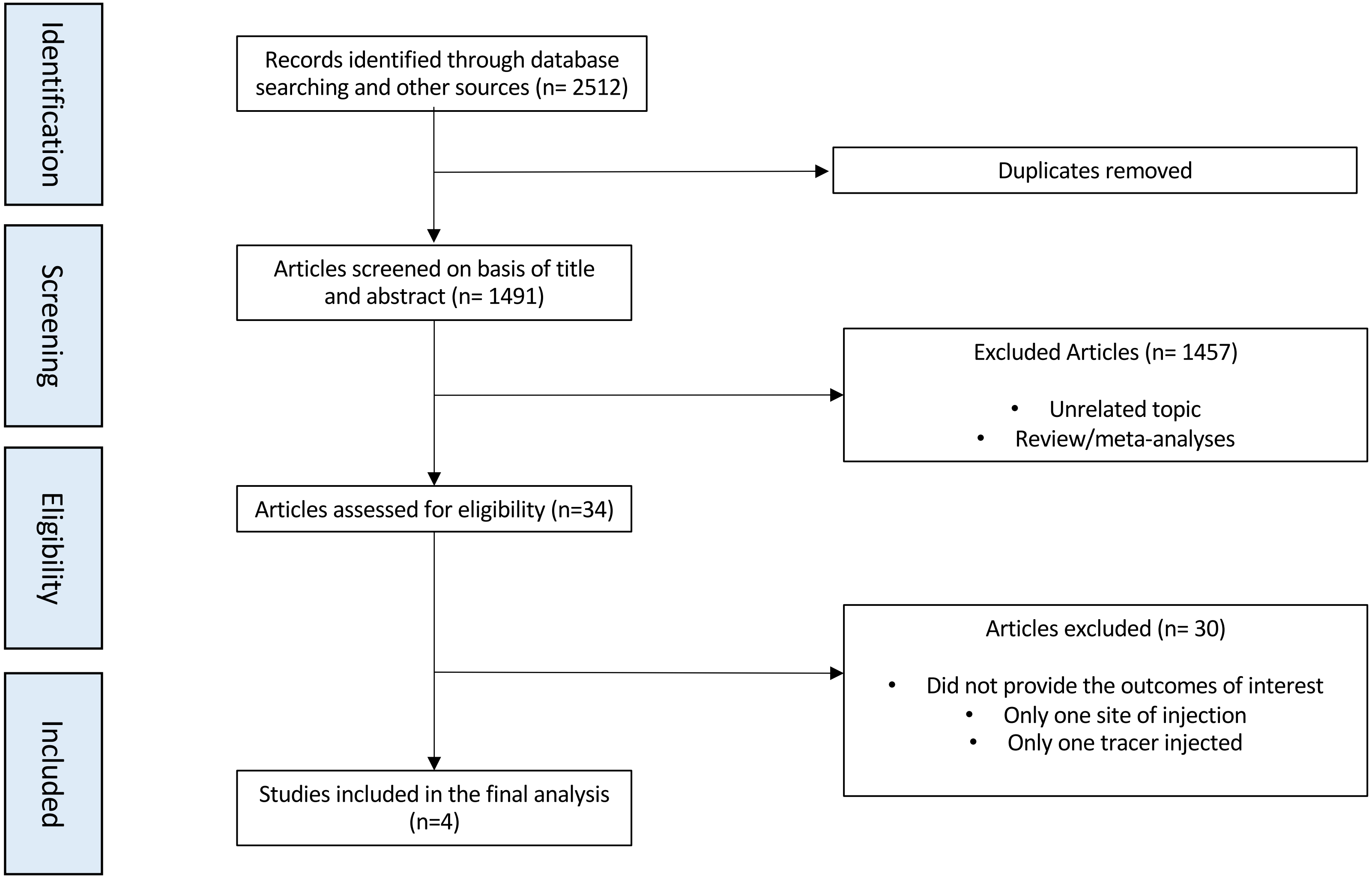

From: Moher D, Liberati A, Tetzlaff J, Altman DG, The PRISMA Group (2009). Preferred Reporting Items for Systematic Reviews and Meta-Analyses: The PRISMA Statement. PLoS Med 6(7): e1000097. doi:10.1371/journal.pmed1000097

For more information, visit www.prisma-statement.org.
