# Supplementary material for: Does the Uterine Injection Site Matter for the Pelvic Sentinel Lymph Node Mapping? A Systematic Review and Meta-Analysis
Source: Medicina (Kaunas). 2025 Apr 10;61(4):699. doi: 10.3390/medicina61040699 (PMC12028796; doi:10.3390/medicina61040699)

Figure S2. Risk of Bias assessment with the Modified Quality Assessment of Diagnostic Accuracy Studies tool 2 (QUADAS-2).

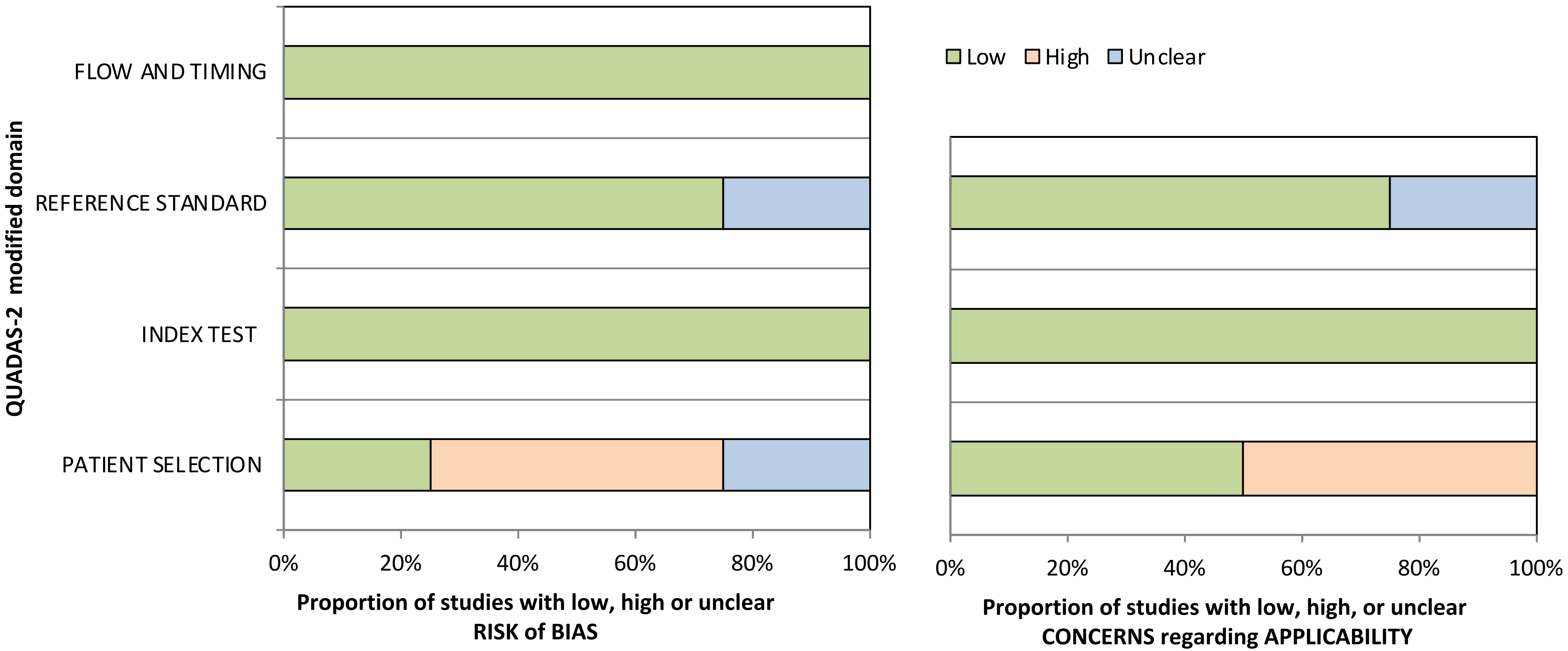

Supplement: Supplementary file 1 [file medicina-61-00699-s001.zip › Figure_S2.pdf]
