# Supplementary material for: Does the Uterine Injection Site Matter for the Pelvic Sentinel Lymph Node Mapping? A Systematic Review and Meta-Analysis
Source: Medicina (Kaunas). 2025 Apr 10;61(4):699. doi: 10.3390/medicina61040699 (PMC12028796; doi:10.3390/medicina61040699)
Supplement: Supplementary file 1 [file medicina-61-00699-s001.zip › Table S1.pdf]

Table S1: Risk of bias assessment for each included study based on the Modified Quality Assessment of Diagnostic Accuracy Studies tool 2 (QUADAS-2)

**Author, year**	**Domain 1: Patient selection**	**Domain 2: Index test**	**Domain 3: Reference standard**	**Domain 4: Flow and timing**
Niikura, 2013	**HIGH**<br>Cases with preoperative lymph nodes suspected of metastasis were excluded	**LOW**	**UNCLEAR**<br>Details are not present for all cases	**LOW**
Sawicki, 2015	**HIGH**<br>Cases with preoperative lymph nodes suspected of metastasis were excluded	**LOW**	**LOW**	**LOW**
Farazestanian, 2019	**UNCLEAR**<br>Patients' selection is not described appropriately	**LOW**	**LOW**	**LOW**
Uccella, 2022	**LOW**	**LOW**	**LOW**	**LOW**
